# Cerebral venous sinus thrombosis associated with *JAK2* V617F mutation-related pre-primary myelofibrosis: a case report and literature review

**DOI:** 10.1186/s12883-024-03913-8

**Published:** 2024-10-12

**Authors:** Jiahao Song, Chanzi Huang, Lina Jia, Mengqi Wang, Chuanjie Wu, Xunming Ji, Haiqing Song, Ran Meng, Da Zhou

**Affiliations:** 1 Department of Neurology, Xuanwu Hospital, Capital Medical University, Beijing, 100053 China; 2 Advanced Center of Stroke, Beijing Institute for Brain Disorders, Beijing, 100053 China; 3 National Center for Neurological Disorders, Xuanwu Hospital, Capital Medical University, Beijing, 100053 China; 4 Department of Neurology, The People’s Hospital of He Chi, Hechi, China

**Keywords:** Cerebral venous sinus thrombosis, Pre-primary myelofibrosis, JAK2 mutation, Case report

## Abstract

**Background:**

Cerebral venous sinus thrombosis (CVST) is a rare but potentially life-threatening subtype of stroke. Prompt and appropriate anticoagulation is crucial for improving the prognosis of CVST and preventing its recurrence. Identifying the underlying cause of CVST is decisive for guiding anticoagulant selection and determining treatment duration.

**Case Presentation:**

A 50-year-old man presented with a 35-day history of headache, nausea, vomiting, and blurred vision. Digital subtraction angiography performed at another facility revealed CVST. A contrast-enhanced black-blood MRI at our center confirmed the diagnosis, which was supported by a high intracranial pressure of 330mmH_2_O. Laboratory tests showed elevated leukocytes and platelet counts, raising suspicion of an underlying myeloproliferative neoplasms (MPNs). A bone marrow biopsy demonstrated increased megakaryocytes and granulocytes, and genetic testing identified the presence of the Janus kinase 2 V617F (JAK2 V617F) mutation, leading to a diagnosis of pre-primary myelofibrosis (pre-PMF). During hospitalization, anticoagulation with nadroparin calcium and fibrinolytic therapy were initiated. Upon discharge, rivaroxaban and aspirin were prescribed to prevent CVST recurrence and arterial thrombosis.

**Conclusion:**

This case highlights the importance of recognizing dynamic changes in routine blood tests that may link CVST to underlying hematological disorders. The JAK2 mutation is not only associated with MPNs but also increases the risk of thrombosis, including CVST. Further investigation is warranted to better understand the mechanisms by which JAK2 mutations contribute to thrombosis and to explore the potential benefits of JAK2 inhibitors in reducing this risk.

**Supplementary Information:**

The online version contains supplementary material available at 10.1186/s12883-024-03913-8.

## Introduction

Cerebral venous sinus thrombosis (CVST) is a rare but fatal form of stroke, representing approximately 1% of all stroke cases [1]. CVST predominately affects young adults, with females being 1.5 to 5 times more susceptible than males [2, 3]. Pathologically, thrombus formation in the venous sinuses obstructs cerebral venous outflow, leading to elevated intracranial pressure (ICP). The impaired venous drainage heightens the risk of intracerebral hemorrhage (ICH), which can result in brain tissue damage and neurological deficits [4]. In severe cases, unchecked ICP can cause brain herniation and death [5]. Observational studies report CVST mortality rates ranging from 6 to 10%. In the acute phase, timely anticoagulation, endovascular thrombectomy, and dehydration are critical to improving prognosis [6].

Anticoagulation therapy remains the cornerstone of CVST treatment, with the duration tailored based on the underlying etiology. For transient thrombogenic conditions, such as glucocorticoid use, major surgery, immobilization, and acute infections, anticoagulation is typically recommended for three to six months [7]. However, in cases of hereditary thrombophilia [8], autoimmune diseases [9], or hematological conditions like polycythemia vera, lifelong anticoagulation may be required to prevent recurrence [10, 11]. Additional factors, such as iron deficiency anemia, can also induce a hypercoagulable state that persists until corrected. Thus, the duration of anticoagulation therapy for CVST must be individualized based on the resolution of the condition [12]. Unfortunately, up to 30–35% of CVST cases have undetermined etiologies, making it challenging yet essential for clinicians to identify potential causes [2].

Primary myelofibrosis (PMF), a clonal disorder of hematopoietic stem cells (HSCs), is characterized by the excessive proliferation of megakaryocytes and granulocytes, ultimately contributing to bone marrow fibrosis and extramedullary hematopoiesis [13, 14]. Approximately 50–60% of PMF cases are driven by the Janus kinase 2 V617F (JAK2 V617F) mutation [15, 16]. Thrombosis, particularly arterial and venous events, is relatively common in PMF [17, 18]; however, CVST is exceedingly rare, accounting for fewer than 1 in 200 thrombotic events in these patients [19]. This article presents a case of CVST in a patient with pre-PMF and a JAK2 V617F mutation, providing insight into the diagnostic and therapeutic challenges posed by this rare association. This case is reported according to CARE guidelines.

## Case presentation

**Fig. 1:**
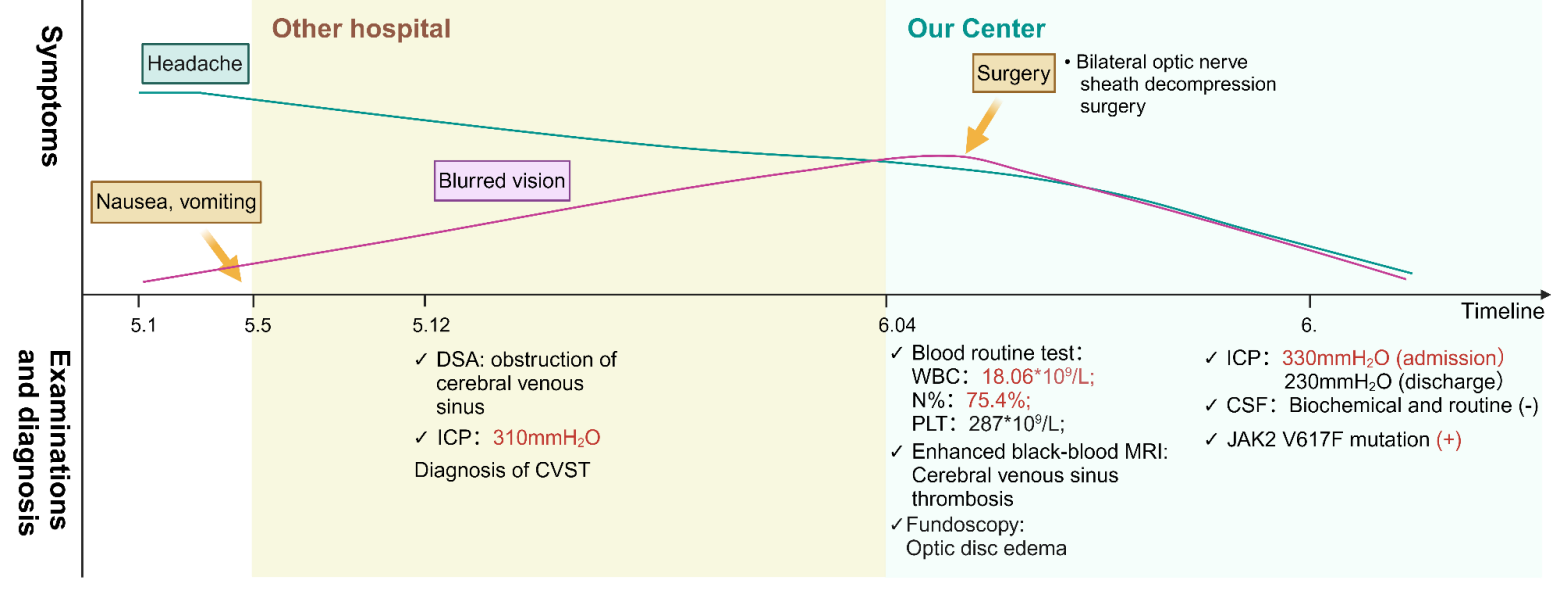
Timeline of this case

A 50-year-old male presented with a 35-day history of diffuse, throbbing headaches, accompanied by nausea, vomiting, and blurred vision. The patient had no prior history of smoking, obesity, or notable medical conditions. His symptoms escalated during the first four days, during which he experienced intermittent nausea and vomiting. Initial investigations at a previous hospital, including digital subtraction angiography (DSA), confirmed the diagnosis of CVST, which was further supported by a lumbar puncture indicating elevated ICP of 310 mmH_2_O (normal range: 80–180 mmH_2_O) (Fig. 1).

**Fig. 2:**
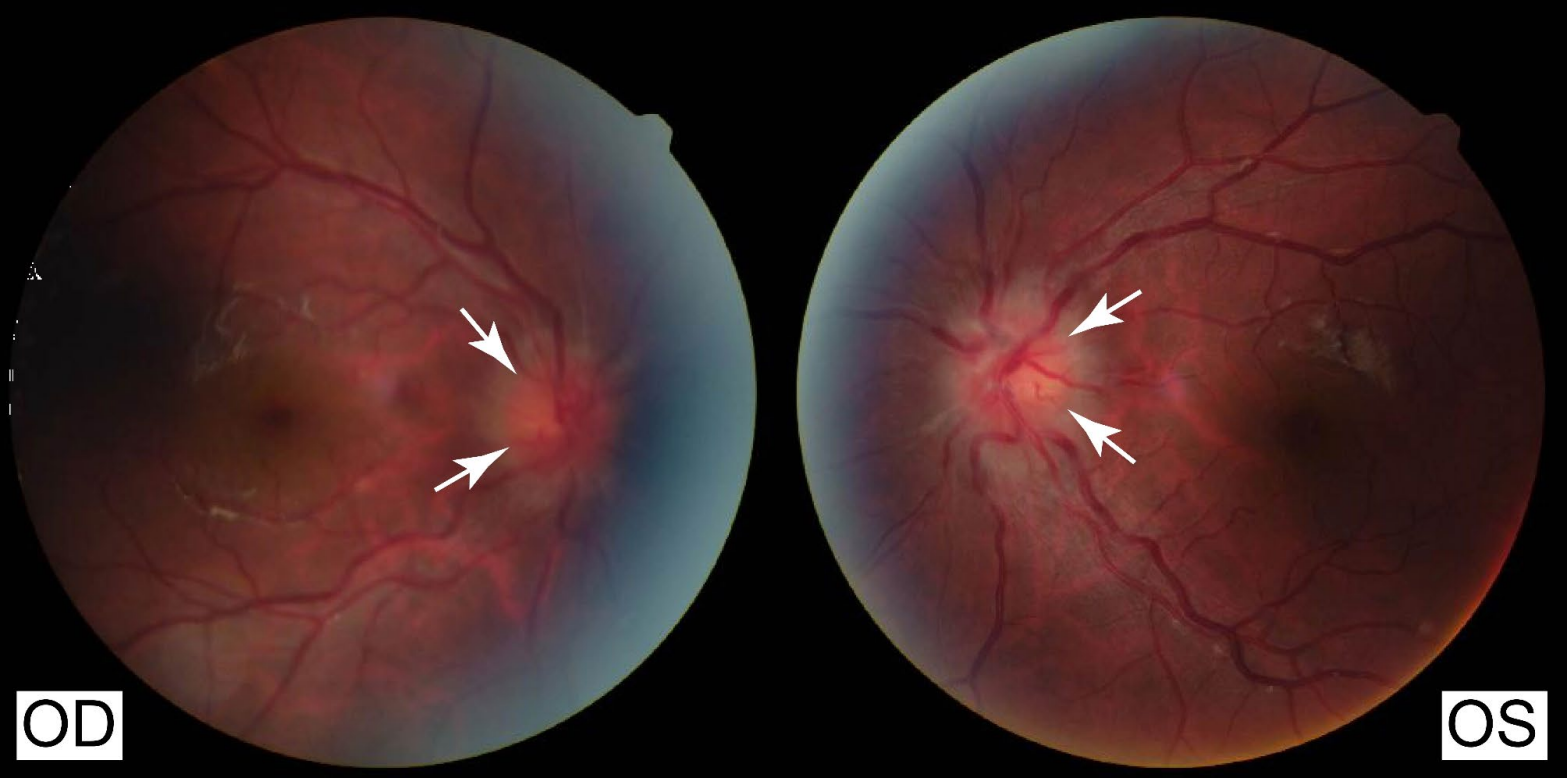
Color fundus photography showing bilateral optic disc edema (Frisén Grade: 3). Abbreviations: OS = oculus sinister; OD = oculus dexter

Despite receiving treatment with aspirin and clopidogrel at the local hospital, the patient continued to experience intermittent episodes of blurred vision. He was subsequently referred to our center for further evaluation of his deteriorating vision and comprehensive management of CVST. Upon admission, ophthalmic examination indicated optic disc edema (Frisén Grade 3) (Fig. 2). Neurological examination revealed declining visual acuity in both eyes and positive neck resistance.

**Fig. 3:**
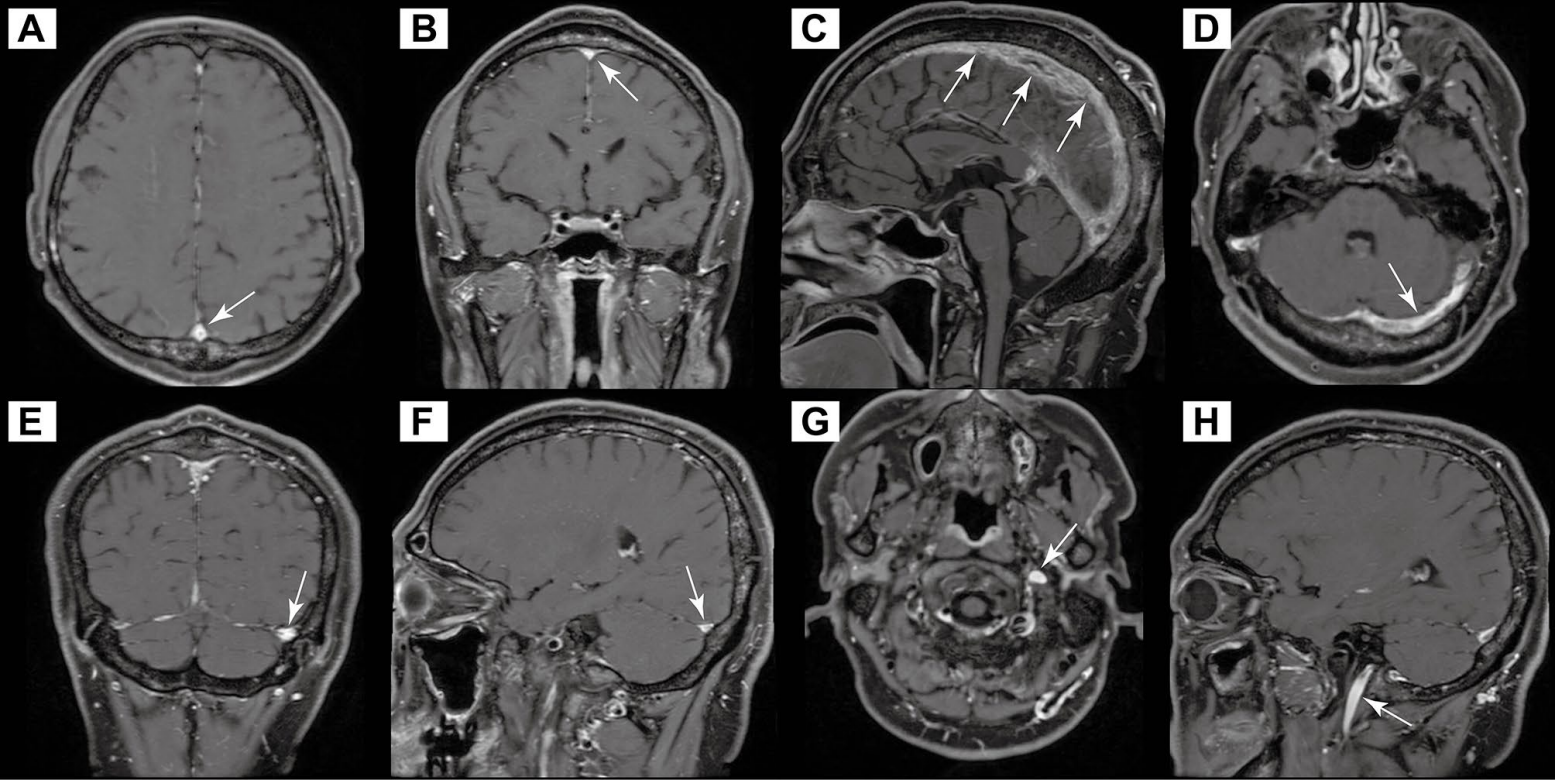
Contrast-enhanced black-blood MRI of the head indicating thrombosis in the superior sagittal sinus (**A**-**C**), left transverse and sigmoid sinus (**D**-**F**), and the upper segment of the left internal jugular vein (**G**-**H**)

Imaging studies at our center, including contrast-enhanced black-blood MRI, confirmed extensive thrombosis involving the superior sagittal sinus, straight sinus, confluence of sinuses, bilateral lateral sinuses, and upper internal jugular veins, as well as cortical veins (Fig. 3). A repeat lumbar puncture showed further elevated ICP (330mmH_2_O, normal range: 80–180 mmH_2_O). Given the risk of further optic nerve damage, bilateral optic nerve sheath decompression surgery was performed to protect the optic nerves and preserve vision.

Routine blood tests demonstrated an elevated leukocyte count (*N* = 18.06 × 10^9^/L, normal range: 4–10 × 10^9^/L) and neutrophil percentage (75.4%, normal range: 50-75%). However, coagulation profiles remained within normal limits, and there were no signs of infection (Supplementary Table 1). These findings, along with previous blood abnormalities (Supplementary Fig. 1), raised suspicion of an underlying hematologic disorder. Hematology consultation suggested the possibility of myeloproliferative neoplasms (MPNs), prompting additional diagnostic workup.

**Fig. 4:**
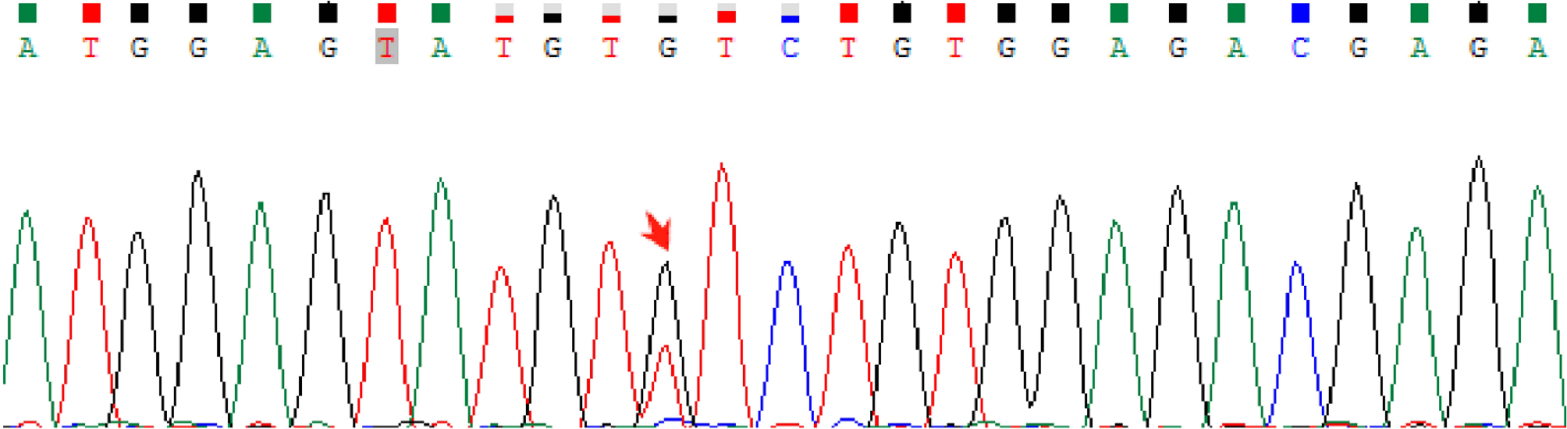
Results of genetic tests for driving genes of myeloproliferative neoplasms in peripheral blood cells

**Fig. 5:**
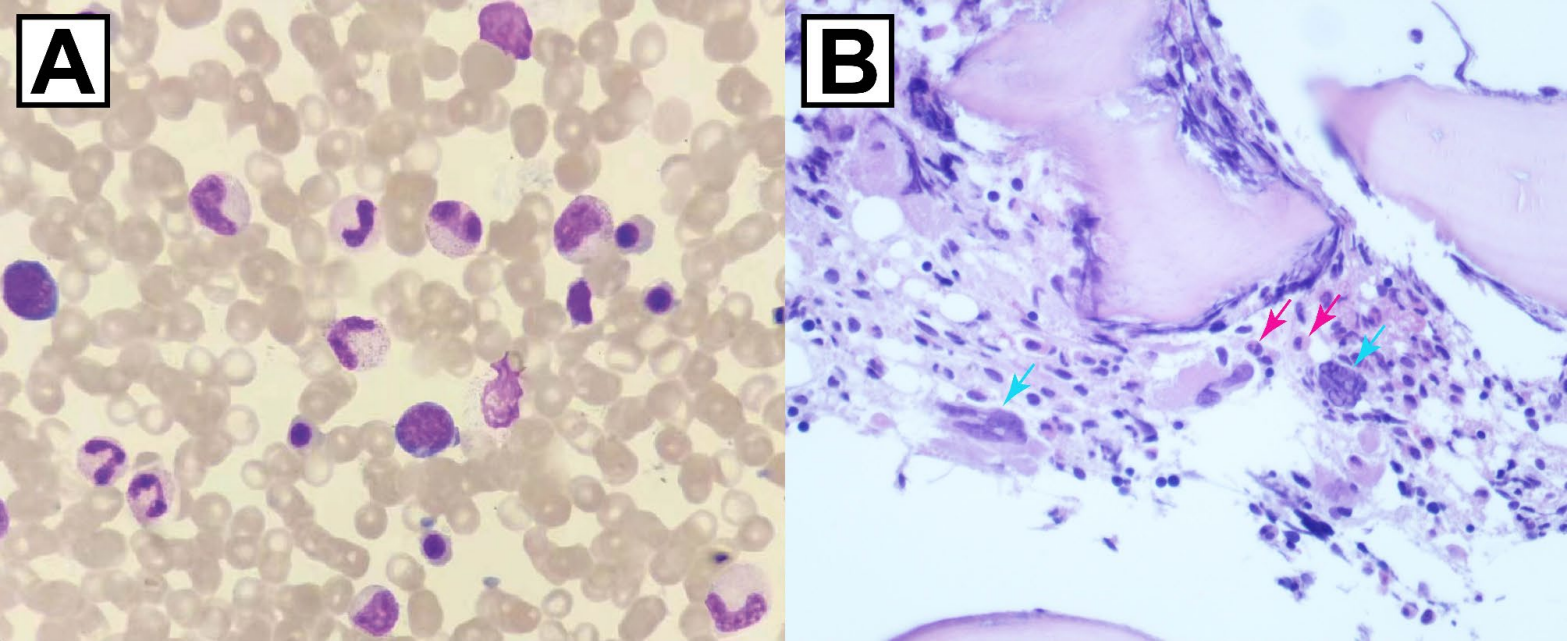
Bone marrow biopsy supporting the diagnosis of pre-PMF. (**A**) Bone marrow cytomorphology indicated a myeloproliferative state. (**B**) Bone marrow biopsy indicated active proliferation of granulocytes (red arrow) and megakaryocytes (blue arrow). Abbreviation: pre-PMF = pre-primary myelofibrosis

The patient’s serum lactate dehydrogenase (LDH) levels were elevated (288 IU/L, normal range: 120–250 IU/L), and genetic testing confirmed the presence of a JAK2 V617F mutation (Table 1 and Fig. 4). Bone marrow biopsy revealed megakaryocytic and granulocytic proliferation, consistent with pre-PMF, according to the 2022 WHO diagnostic criteria (Fig. 5). Notably, BCR-ABL fusion gene testing ruled out chronic myeloid leukemia, and no other driver or non-driver gene mutations were detected. The bone marrow karyotype screening detected no chromosomal abnormalities. Additional tests for potential thrombosis causes, including infections, hereditary thrombophilia, and autoimmune disorders, were negative (Table 2).

**Table 1:** Genetic tests for driving genes of myeloproliferative neoplasms in both peripheral blood cells and bone marrow

Gene name	Testing items	Location	Results
JAK2	Exon 14	Peripheral blood	NM_004972.4:c.1849G > T (p.Val617Phe)
	Exon 12		None
MPL	Exon 10		None
CALR	Exon 9		None
JAK2	Exon 14	Bone marrow	NM_004972.4:c.1849G > T (p.Val617Phe)
	Exon 12		None
MPL	Exon 10		None
CALR	Exon 9		None

Abbreviation: JAK2 = Janus Kinase 2; MPL = Myeloproliferative Leukemia Virus Oncogene; CALR = Calreticulin

**Table 2:** Results of other potential risk factors for CVST

Category	Items	Results	Reference range
Infection	CSF pathogen analysis	Negative	Negative
Hereditary thrombophilia	Homocysteine	13.5 umol/L	0–20 umol/L
	Thrombophilia gene hotspot testing*	Negative	Negative
	IgA	2.29 g/L	0.82–4.53 g/L
	IgM	1.08 g/L	0.46–3.04 g/L
	IgG	14.50 g/L	7.51–15.6 g/L
	IgE	50.20 IU/ml	5-165 IU/L
	Complement C3	0.99 g/L	0.79–1.52 g/L
	Complement C4	0.21 g/L	0.16–0.38 g/L
	Rheumatoid factor	Negative	Negative
	Antinuclear antibody spectrum	Negative	Negative
	Lupus anticoagulant	Negative	Negative
	Anti-neutrophil cytoplasmic antibodies	Negative	Negative
	Anticardiolipin antibodies	Negative	Negative
Cancer	Tumor markers	Negative	Negative
	Paraneoplastic antibody spectrum (serum&CSF)	Negative	Negative
	Chest CT scan	No signs for tumor	Negative
	Abdominal CT scan	No signs for tumor+	Negative

*: Nine genes tested: PROC, PROS1, SERPINC1, F2, F5, HRG, THBD, PAI-1, and MTHFR

+: No signs for other tumors except for MPNs

Abbreviation: CVST = Cerebral venous sinus thrombosis; CSF = Cerebrospinal fluid; Ig = Immunoglobulin. MPNs = Myeloproliferative neoplasms

**Fig. 6:**
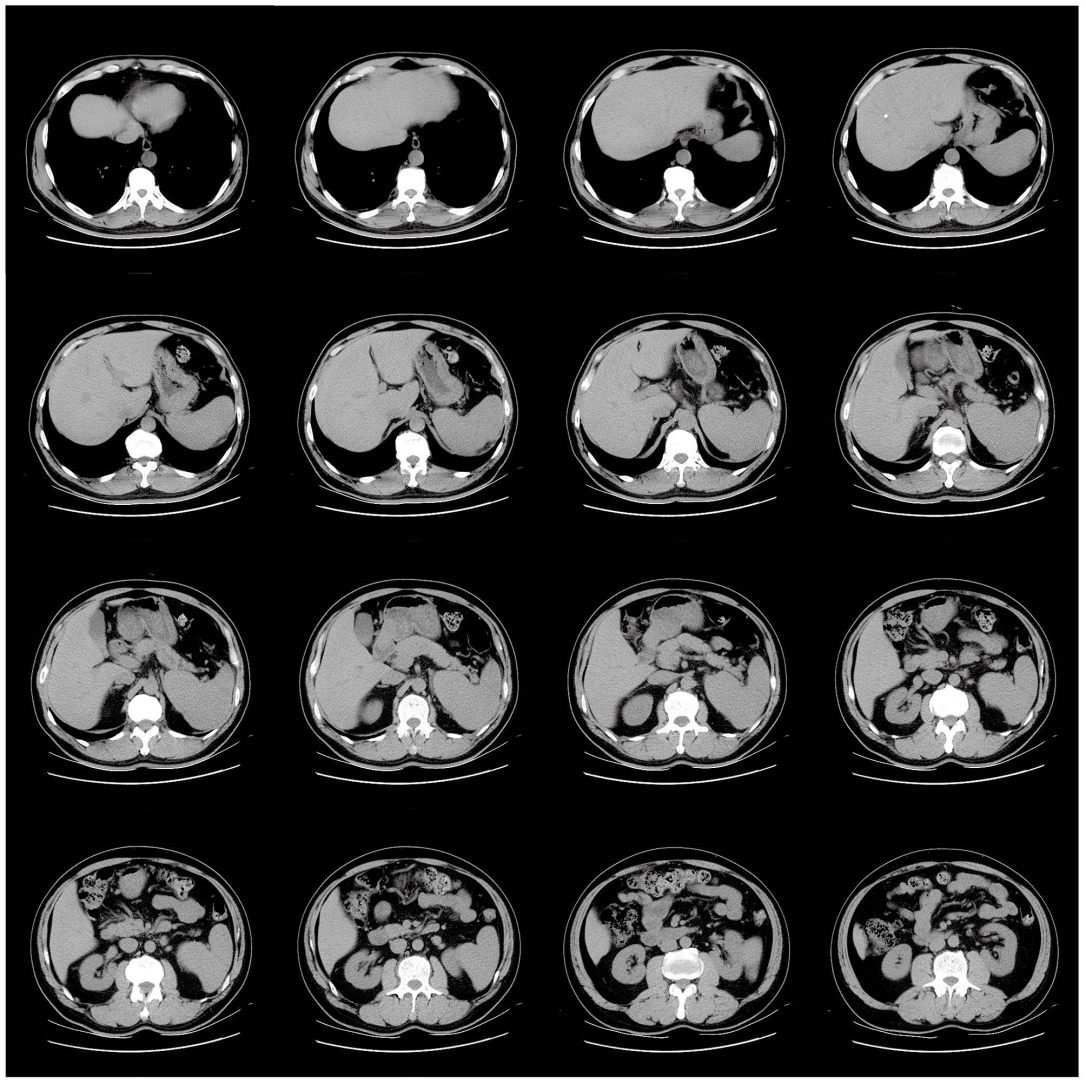
Abdominal CT scan: hepatic calcified foci and splenomegaly

Upon admission, the patient was started on standardized anticoagulation (Nadroparin calcium, 0.8 ml q12h), along with fibrinolytic (Batroxobin) and dehydration therapies. His ICP gradually decreased to 230 mmH₂O, and his headache symptoms improved. To reduce the patient’s splenomegaly (Fig. 6) and to improve long-term prognosis, ruxolitinib was administered. After 16 days of hospitalization, he was discharged with instructions to continue rivaroxaban and aspirin for preventing CVST recurrence and arterial thrombosis. He was referred to the hematology department for further management of pre-PMF.

At the two-month follow-up, the patient reported complete resolution of symptoms, including headaches and blurred vision, with no complications or discomfort.

## Discussion

This report presents a rare case of CVST in association with pre-PMF and the JAK2 V617F mutation, a combination that remains under-researched. According to the 2022 WHO diagnostic criteria, pre-PMF represents an early stage of PMF, marked by megakaryocyte and granulocyte proliferation, but without the significant bone marrow fibrosis characteristic of overt PMF [20].Clinically and morphologically, pre-PMF shares similarities with essential thrombocythemia (ET), with thrombosis being a recognized complication of both disorders.

Identifying the underlying cause of CVST is essential for determining appropriate management and prognosis. Initially, hematological disorders were not considered a primary differential diagnosis in this patient due to the lack of constitutional symptoms (e.g., fatigue, fever, and weight loss) or other typical signs of MPNs. Elevated leukocyte and platelet counts were first attributed to a stress response. However, persistent abnormal blood results, combined with splenomegaly identified on CT, and negative results for alternative causes such as infections, thrombophilia, and autoimmune diseases, pointed toward a hematologic origin. This is an important reminder that such findings can often be overlooked in clinical practice. Similar to other MPN subtypes, pre-PMF carries a high risk of thrombosis. A prospective cohort study from Taiwan reported thrombosis in approximately 20% of pre-PMF patients, with cerebral ischemic stroke being the most common manifestation [21, 22]. Pre-PMF patients were found to have higher rates of splanchnic vein thrombosis compared to those with ET.

The JAK2 V617F mutation, a missense mutation replacing valine with phenylalanine at amino acid position 617 within the pseudokinase domain of the JAK2 protein, is the predominant genetic aberration in MPNs and is detected in 50-60% of PMF patients. This mutation has a significant impact on the clinical phenotype, complications, and prognosis of MPNs. PMF patients harboring JAK2 mutations often exhibit higher leukocyte counts and hemoglobin levels and are more prone to thrombosis [16]. A national cohort study involving 302 individuals across 17 hospitals linked the JAK2 V617F mutation with an increased risk of thrombosis and a higher likelihood of progression to MF and leukemia in ET patients [23]. Furthermore, a multicenter cohort study of 74 participants revealed a 91% detection rate of JAK2 mutation in MPNs-associated CVST, significantly higher than in the general MPN population [24]. These findings underscore the critical role of the JAK2 mutation in the development of CVST in MPN patients.

JAK2, an intracellular tyrosine kinase, serves as a key downstream signal transducer for various growth hormones. When activated by cytokines such as erythropoietin, thrombopoietin, and interleukin-3, JAK2 phosphorylates tyrosine residues on receptors, creating docking sites for STAT3 and STAT5. The phosphorylated STATs then dimerize and translocate into the nucleus, where they modulate transcription to promote the survival and proliferation of HSCs [25]. In HSCs with JAK2 mutations, there is an overproduction of activated leukocytes and platelets, leading to the release of excessive reactive oxygen species, pro-inflammatory cytokines, and thromboxane, all of which collectively increase the risk of arterial and venous thrombosis [26]. Interestingly, this patient’s peripheral blood showed no evidence of a chronic inflammatory state, as indicated by normal levels of C-reactive protein, interleukin-6, and erythrocyte sedimentation rate, an observation that warrants further investigation and dynamic monitoring. CVST is a rare complication in MPN patients. A prospective cohort study of 2,143 MPN individuals documented nine cases of CVST (0.4%), with three occurring before, three during, and three after the diagnosis of MPNs [19]. Given our center’s role as the largest facility for cerebral venous disorders in China, it may be prudent to screen all CVST patients who exhibit significant and/or persistent abnormalities in peripheral blood counts for MPNs and JAK2 mutations, particularly in cases where no other clear cause of CVST is identified. Additional clinical features, such as splenomegaly, and constitutional or systemic symptoms—such as fatigue, unintended weight loss, night sweats, and fever—should also raise suspicion for MPNs. Early detection through this approach could facilitate timely diagnosis, potentially even before overt MPN symptoms develop.

Negative BCR-ABL fusion gene testing ruled out Philadelphia chromosome-positive chronic myeloid leukemia. Subsequently, we conducted a comprehensive next-generation sequencing panel for myeloid malignancies, which confirmed the presence of only the JAK2 V617F mutation, with no other driver or non-driver gene mutations detected. Although driver genes are typically mutually exclusive, they can co-mutate with non-driver genes, potentially influencing prognosis. No abnormal karyotypes were identified. Currently, a comprehensive molecular prognostic assessment model (MIPSS-70 + Version 2.0) for PMF integrates clinical manifestations (degree of anemia and systemic symptoms), gene mutations (mutation of driver and non-driver genes), and karyotype to develop personalized treatment plans [27, 28]. For high- or very-high-risk PMF, allogeneic HSC transplantation is recommended. JAK2 inhibitors, such as ruxolitinib, are employed to block the overactivated JAK-STAT signaling pathway, which plays a central role in PMF pathogenesis. Although prognostic systems like MIPSS-70 + Version 2.0 may not be applicable to this patient due to the focus on overt PMF, the presence of CVST indicates a substantial thrombotic burden requiring a more tailored therapeutic approach. While JAK2 inhibitors are typically used for symptomatic management in PMF, their potential to reduce thrombotic risk, especially in patients with significant thrombotic events like CVST, may justify their use even in the absence of constitutional symptoms. Given the unique risks in this case, long-term follow-up is crucial to monitor disease progression and manage potential thrombotic events. Further research is necessary to establish evidence-based guidelines for the management of pre-PMF patients with thrombotic complications, as current data is limited.

Anticoagulation therapy remains the cornerstone of CVST treatment, with emerging evidence supporting the use of fibrinolytic therapy to improve venous sinus recanalization [29, 30]. In this case, the patient received nadroparin calcium and batroxobin, leading to a reduction in ICP and improvement in symptoms. Given the persistent risk of thrombosis in pre-PMF, lifelong anticoagulation with rivaroxaban was prescribed to prevent CVST recurrence, and aspirin was added for arterial thrombus prevention. Regular monitoring of complete blood count, coagulation parameters, factor Xa activity, and signs of systemic bleeding is critical to assess bleeding risk. Cytoreductive therapy was not initiated, as the patient’s hematocrit levels remained below 45%. The patient was advised to continue with specialized pre-PMF management under hematology. Currently, no high-level, evidence-based guidelines exist for treating MPNs-associated CVST. Several key areas, including clinical characteristics, prognostic features, and risk stratification of MPNs-associated CVST, require further exploration. Moreover, the safety and efficacy of novel oral anticoagulants in thrombosis prevention, and the potential of JAK2 inhibitors like ruxolitinib to reduce thrombotic risk warrant continued clinical evaluation.

## Conclusion

This study presents a rare case of pre-PMF with a JAK2 V617F mutation leading to CVST. CVST occurrence in MPN patients is rare, sometimes preceding or coinciding with the diagnosis of MPNs. Recognizing dynamic changes in routine blood tests is crucial for attributing hematologic disease-associated CVST. The JAK2 mutation, a key driver gene for MPNs, not only relates to MPN development but also increases the risk of thrombosis, including CVST. Further research into the underlying mechanisms and the potential role of JAK2 inhibitors in reducing thrombotic risk is of great significance.

## Electronic supplementary material

Below is the link to the electronic supplementary material.

Supplementary Material 1: Figure 1. Number of white blood cells and platelets, and the proportion of neutrophils from tests conducted at other hospitals and our center. The red arrow indicated that the patient visited our center 35 days after the onset of headaches.

Supplementary Material 2: Table 1. Body temperature and three inflammatory indicators.

## Data Availability

No datasets were generated or analysed during the current study.

## Abbreviations

CVST: Cerebral venous sinus thrombosis
ICP: Intracranial pressure
ICH: Intracerebral hemorrhage
PMF: Primary myelofibrosis
HSCs: Hematopoietic stem cells
DSA: Digital subtraction angiography
MPNs: Myeloproliferative neoplasms
LDH: Lactate dehydrogenase
ET: Essential thrombocythemia
PV: Polycythemia vera
