# Supplementary material for: Cerebral venous sinus thrombosis associated with JAK2 V617F mutation-related pre-primary myelofibrosis: a case report and literature review
Source: BMC Neurol. 2024 Oct 12;24:386. doi: 10.1186/s12883-024-03913-8 (PMC11470542; doi:10.1186/s12883-024-03913-8)

**Supplementary Figure 1.** Number of white blood cells and platelets, and the proportion of neutrophils from tests conducted at other hospitals and our center. The red arrow indicated that the patient visited our center 34 days after the onset of headaches.


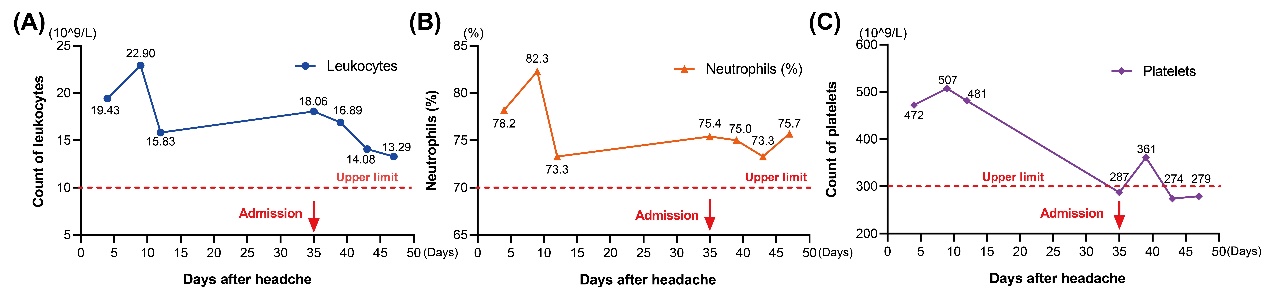

Supplement: Supplementary file 1 — Supplementary Material 1: Figure 1. Number of white blood cells and platelets, and the proportion of neutrophils from tests conducted at other hospitals and our center. The red arrow indicated that the patient visited our center 35 days after the onset of headaches. [file 12883_2024_3913_MOESM1_ESM.docx]
