# Supplementary material for: Cerebral venous sinus thrombosis associated with JAK2 V617F mutation-related pre-primary myelofibrosis: a case report and literature review
Source: BMC Neurol. 2024 Oct 12;24:386. doi: 10.1186/s12883-024-03913-8 (PMC11470542; doi:10.1186/s12883-024-03913-8)
Supplement: Supplementary file 2 — Supplementary Material 2: Table 1. Body temperature and three inflammatory indicators. [file 12883_2024_3913_MOESM2_ESM.docx]

**Supplementary Table 1.** Body temperature and three inflammatory indicators

| **Indicator** | **Results** | **Reference** |
| --- | --- | --- |
| Body temperature | 36.5℃ | 36-37℃ |
| C-reactive protein | 0.52mg/L | 0-3mg/L |
| Interleukin-6 | 3.36pg/ml | <7.0pg/ml |
| Erythrocyte sedimentation rate | 2mm/h | 0-15mm/h |
